# Anastrozole for the prevention of breast cancer in high-risk postmenopausal women: cost-effectiveness analysis in the UK and the USA

**DOI:** 10.1186/s12913-024-10658-0

**Published:** 2024-02-13

**Authors:** XiaoXia Wei, Jiaqin Cai, Huiting Lin, Wenhua Wu, Jie Zhuang, Hong Sun

**Affiliations:** 1 Department of Pharmacy, Shengli Clinical Medical College of Fujian Medical University, Fujian Provincial Hospital, No. 134, East Street, Gulou District, Fuzhou, 350001 P. R. China; 2 School of Pharmacy, Fujian Medical University, No. 1 Xuefu North Road, University Town, Fuzhou, 350122 P. R. China

**Keywords:** Anastrozole, Breast cancer, High risk, Cost-effectiveness, Prevention

## Abstract

**Purpose:**

The effectiveness of anastrozole for breast cancer prevention has been demonstrated. The objective of this study was to evaluate the cost-effectiveness of anastrozole for the prevention of breast cancer in women with a high risk of breast cancer and to determine whether anastrozole for the primary prevention of breast cancer can improve the quality of life of women and save health-care resources.

**Methods:**

A decision-analytic model was used to assess the costs and effects of anastrozole prevention *versus* no prevention among women with a high risk of breast cancer. The key parameters of probability were derived from the IBIS-II trial, and the cost and health outcome data were derived from published literature. Costs, quality-adjusted life-years (QALYs), and incremental cost-effectiveness ratios (ICERs) were calculated for the two strategies,One-way and probabilistic sensitivity analyses were performed.

**Results:**

In the base case, the incremental cost per QALY of anastrozole prevention was £125,705.38/QALY in the first 5 years compared with no prevention in the UK, above the threshold of WTP (£3,000/QALY),and in the 12-year period, the ICER was £8,313.45/QALY, less than WTP. For the US third-party payer, ICER was $134,232.13/QALY in the first 5 years and $8,843.30/QALY in the 12 years, both less than the WTP threshold ($150,000/QALY).

**Conclusion:**

In the UK and US, anastrozole may be a cost-effective strategy for the prevention of breast cancer in high-risk postmenopausal women. Moreover, the longer the cycle of the model, the higher the acceptability. The results of this study may provide a scientific reference for decision-making for clinicians, patients, and national medical and health care government departments.

## Introduction

In recent years, with in-depth research into breast cancer pathogenesis, breast cancer treatment has become increasingly personalised and 5-year survival rates has increased annually [1]. However, as the incidence of breast cancer continues to rise, the burden of breast cancer is intensifying. Breast cancer has now surpassed lung cancer as the world's most common cancer, according to the latest global cancer data for 2020, and is a major threat to women's health worldwide. Many high-income countries, including the United Kingdom(UK) and the United States(USA), are facing the burden of disease for the growing number of breast cancer patients [2].

Breast cancer also negatively affects the economic growth of countries, with an enormous economic cost of $2.0 trillion in international dollars (INT) [3]. Primary preventive care for breast cancer is urgently needed to reduce the huge burden of breast cancer, including screening for risk factors, lifestyle modifications, risk-reducing surgery, and drug prevention [4]. However, breast cancer prevention efforts are not used effectively and cannot keep up with breast cancer treatment [5].

In 1998, the selective oestrogen receptor modulator tamoxifen became the first FDA-approved drug for breast cancer prevention, especially in premenopausal patients and those with dysplasia. In 2007, the FDA approved the preventive effect of raloxifene in postmenopausal women [6]. However, tamoxifen and raloxifene increase the risk of thromboembolism and endometrial cancer, so many doctors are reluctant to prescribe drug prophylaxis. This is also a concern for patients and a reason for them to stop taking the drugs. Studies have shown that the uptake of therapeutic agents for the prevention of breast cancer is low, and long-term persistence is often insufficient for women. Therefore, the prevention of tamoxifen or raloxifene has not been widely used in clinical practice [7].

Oestradiol is an important carcinogen of breast cancer, and aromatase can convert oestrogen into oestradiol, which has an important catalytic effect on oestradiol production, so reducing the level of oestradiol can reduce the risk of breast cancer [8]. Anastrozole, a third-generation aromatase inhibitor, has been used for more than 20 years to treat postmenopausal women with oestrogen receptor-positive breast cancer by blocking oestrogen production in the body [9]. By 2003, the International Breast Cancer Intervention Study II (IBIS-II) began to explore the preventive value of anastrozole in postmenopausal women at high risk of breast cancer and to evaluate its safety and efficacy in the primary prevention of breast cancer. The five years result showed that anastrozole reduced the risk of aggressive oestrogen receptor-positive breast cancer in postmenopausal high-risk women without new toxic side effects and may become the drug of good choice for prevention in postmenopausal women at high risk for breast cancer [10]. Anastrozole was included in the UK and US breast cancer drug prevention guidelines in 2017 and 2019. The recently published long-term follow-up results of the IBIS-II trial showed a significant reduction in the incidence of breast cancer in women 7 years after discontinuation of anastrozole (49%), equivalent to every 29 women taking anastrozole for 5 consecutive years, and one case of breast cancer can be prevented in 12 years [11]. However, as a drug, anastrozole is costly and may have adverse reactions during its use. For this reason, it is necessary to return to the outpatient clinic for regular check-ups and follow-ups. This means that to prevent one case of breast cancer in 12 years, 29 women at risk would need to take anastrozole for five years. Is this "value for money", or does it take up medical resources and cause extra burden for patients? Can it be supported at a time of rising health care costs?

This information is important to the third-party payers, the organization that pays the bills for a patient's health care, as well as to the general public. An important next step is to conduct a cost-effectiveness analysis to assess the potential costs and health outcomes to provide the data needed to advocate for anastrozole prevention in women. Therefore, this study intends to evaluate the economic value of anastrozole for the prevention of breast cancer in women with high risk from the third payer’s perspective in the UK and the USA.

## Materials and methods

### Study subjects

For mathematical simulations, the construction of an economic model requires the collection of the probability of occurrence of relevant events. So the key clinical parameters, such as breast cancer incidence, death, other cancers, and the rate of major adverse events were derived from the long-term results of anastrozole for breast cancer prevention (IBIS-II) [11]. IBIS-II is an international, randomized, double-blind, placebo-controlled trial that included 153 breast cancer treatment centres across 18 countries, such as the United Kingdom, the United States, Switzerland, Germany, France, etc. The researchers recruited postmenopausal women aged 40–70 years who were at increased risk of breast cancer. Exclusion criteria were premenopausal, previous breast cancer (including ductal carcinoma in situ) diagnosed more than 6 months before the start of the trial, current or previous use of tamoxifen, raloxifene or other selective oestrogen receptor modulator (SERM) for more than 6 months, participated in IBIS-I [10], intended to continue oestrogen-based hormone replacement therapy unless on at least 5 years of off-trial treatment, or had previously performed or planned to undergo prophylactic mastectomy. Patients with the above criteria were the population simulated by our economic model.

### Treatment strategies

Base on the IBIS-II trail, the treatment decisions in our model were receiving anastrozole (1 mg daily, orally) or the equivalent placebo for a 5-year treatment period, with annual follow-up, during which the experiment was carried out by laboratory examination and imaging evaluation. After treatment, women continued to be followed annually to collect data on the incidence, death, other cancers, and major adverse events (cardiovascular and fractures), with the main outcome being breast cancer, including the treatment period. A total of 12 years of follow-up was carried out [11].

### Model establishment

**Fig. 1:**
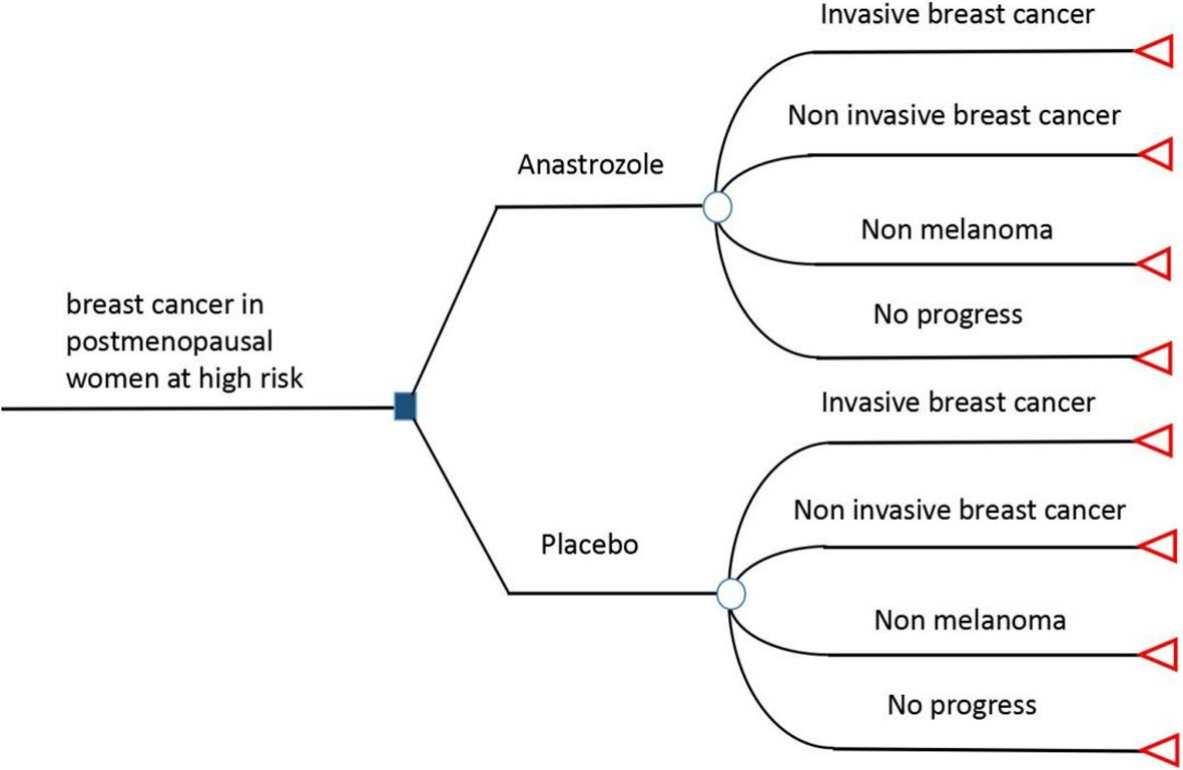
The decision-analytic model

In this study, from the perspective of third-party payers in the UK and the USA, a decision-analytic model was developed by using TreeAge Pro 2023 (TreeAge Software LLC., Williamstown, MA, USA) to evaluate costs and health outcomes of anastrozole or placebo for breast cancer prevention in high-risk postmenopausal women. Because the IBIS-II study pointed out that during the 12-year follow-up, among those who died, the main cause of death was other cancers, cardiovascular or unknown, and only a very small proportion of deaths were due to breast cancer, we cannot accurately obtain the transition probability from the progression state of the disease to the death state. The Markov model was not applicable, so we chose the decision-analytic model. At the same time, the follow-up results of the IBIS-II study found that prophylactic use of anastrozole significantly reduces the incidence of non-melanoma tumours in addition to reducing the incidence of breast cancer.There was no significant difference in the incidence of other cancers between the two groups. Therefore, referring to the outcome of the IBIS-II study, we divided the progression status of this model into four statuses: invasive breast cancer, noninvasive breast cancer, nonmelanoma, and without progression (Fig. 1). The probability parameters of relevant events were input into the model. According to the follow-up results of the IBIS-II study, the model was established with a cycle period (cycle) of 1 year and operation periods (time horizon) of 5 years and 12 years. This study followed the Consolidated Health Economic Evaluation Reporting Standards (CHEERS) reporting guidelines [12].

### Model assumptions

According to the IBIS-II trial [10], there was no significant difference in adverse events between the anastrozole group and the placebo group, so we did not consider the cost and utility of adverse reactions in the model. In addition, we used the same nonmelanoma incidence data in both run cycles because the study did not specify the time period of nonmelanoma incidence. Since the study did not introduce the follow-up treatment plan for patients diagnosed with invasive breast cancer, noninvasive breast cancer, and nonmelanoma, we set the follow-up-related treatment plan and follow-up plan according to the guidelines and published literature. Patients diagnosed with invasive breast cancer underwent a breast cancer biopsy, eight cycles of chemotherapy followed by surgical resection of breast cancer, semiannual laboratory evaluations and outpatient management fees were paid once, and annual breast cancer imaging evaluations (X + MRI). Patients diagnosed with noninvasive breast cancer were treated with breast cancer surgery without chemotherapy, and the rest of the follow-up plans were the same as those of patients with invasive breast cancer [13, 14]. Patients diagnosed with nonmelanoma underwent a single diagnosis, laboratory evaluation (biopsy and dermatopathology), and surgical resectionr [15, 16]. The follow-up plan for patients without progression is the same as for patients with invasive breast cancer. The progress of each state in the anastrozole group and the placebo group was monitored and treated according to the above protocol.

### Costs and utilities

A cost-effectiveness analysis was perform from the perspective of the third-party payers, so the model only considered direct medical costs. The direct non-medical costs, indirect costs and intangible costs were not covered by health insurance. Costs were calculated as direct medical costs, including drug, examination, surgery, treatment, outpatient management, etc. The unit price of the items was derived from related literature published in the UK and the USA [17–29]. Input costs were reported in 2023 GBP or USD. Where necessary, input costs were adjusted to 2023 GBP or USD using the medical care component of the Consumer Price Index.

The cost of the drug was mainly anastrozole. In calculating dosage amounts of anastrozole was derived from IBIS-II trials(1 mg daily, orally for 5-year treatment period). The costs of disease management include follow-up-related treatment and outpatient follow-up examinations. Patients diagnosed with invasive breast cancer received a breast cancer biopsy, eight cycles of chemotherapy followed by surgical resection, patients diagnosed with noninvasive breast cancer were treated with breast cancer surgery without chemotherapy, patients diagnosed with nonmelanoma underwent a single diagnosis, laboratory assessment (biopsy and dermatopathology), and surgical resection. And all patients received semiannual laboratory assessment, annual breast cancer imaging evaluations (X-ray/MRI), and outpatient management. Combined calculations based on the above probability of occurrence of disease events, frequency of disease management and corresponding unit prices by TreeAge Software. Details of each cost parameter and the range of values are shown in Table 1.

**Table 1:** Costs and utility used in the cost-effectiveness analysis model

Parameter	United Kingdom	United States	Distribution	Source
	**Base(range)**	**Base(range)**		
***cost***				
Anastrozole(per mg)	£3.14(2.512–3.768)	$3.11(2.488–3.732)	Gamma	[17, 18]
Breast cancer Laboratory assessment(twice a year)	£301.06(247.87–377.47)	$420(467.9–1,403.8)	Gamma	[19, 20]
Breast cancer biopsy, one-time	£353.5(171.15–588)	$831(664.8–997.2)	Gamma	[21, 22]
Imaging assessment of breast cancer(per year)(X/MRI)	£88.31(57.69–148.24)	$135(67.5–535)	Gamma	[21, 23]
Outpatient management of breast cancer(twice a year)	£130(104–156)	$203.91(183.52–224.30)	Gamma	[24, 25]
Surgical resection of breast cancer	£4,422(4,340–4,827)	$10,618(8,494.4–12,741.6)	Gamma	[23, 24]
Chemotherapy for breast cancer(per month)	£5,504(4,403.2–6,604.8)	$4,835.85(4,546.29–5,556.57)	Gamma	[25, 26]
Surgical resection of non melanoma	£885(708–1,062)	$2,507.1(1,141.99–16,761.27)	Gamma	[27, 28]
Non-melanoma diagnosis	£224(179.2–268.8)	$2,493(2,397–2,590)	Gamma	[27, 29]
Non-melanoma Laboratory assessment(twice a year)	£327(261.6–392.4)	$45(36–54)	Gamma	[27, 29]
***Health utility value***				
Invasive breast cancer	0.731(0.5848–0.8772)		Beta	[30]
Non invasive breast cancer	0.79(0.632–0.948)		Beta	[30]
Non-melanoma	0.905(0.85–0.95)		Beta	[31]
High risk women	0.99(0.9405–1)		Beta	[32]

This study used quality-adjusted life years (QALYs) as an outcome measure, and health utility values were used to convert one year of survival in a diseased state to one year in a fully healthy state (that is, 1 QALY). The utility value is 1 in the fully healthy state and 0 in the dead state. The health utility values for each state were obtained from published articles on pharmacoeconomics (Table 1) [30–32].

### Cost-effectiveness analysis

Results of the study output included cost, quality-adjusted life year (QALY) and incremental cost-effectiveness ratio (ICER). The ICER results were compared with the willingness to pay (WTP) as a threshold. In pharmacoeconomic analysis, the WTP is a threshold used to assess whether the ICER is acceptable or not. If the ICER is less than the threshold, the intervention is economic compared to the control, and conversely, it is not. For UK payers, the WTP threshold was set at GBP £30,000 according to The National Institute for Health and Care Excellence (NICE) guidance [33], and for USA payers, the WTP threshold was set at $150,000/QALY [34].

### Sensitivity analysis

In the one-way sensitivity analysis, each parameter was varied within a set range, and the effect of each parameter on the results was evaluated. Publicly available data with upper and lower bounds were included, and for those values that were not available, costs were varied within ± 20% of the baseline value, and the value of health utility was varied within ± 10% as a sensitivity analysis. The maximum health utility value is 1. When the value exceeds 1, it is taken as 1. Followed the economic evaluation guidelines in recommending that in Reference Case analyses, costs and health effects should be discounted at the same rate. A discount rate of 3.5% per annum has been used for UK payers [33], and 3% per annum has been used for USA payers, with a range of 0% to 8% used for sensitivity analysis in our mode [35].

The influence of changes within the range on the results and the specific parameter change ranges are shown in Table 1, and the results arranged in the order of the magnitude of the influence of parameter changes on the model results are represented by a storm diagram. The corresponding distributions were set for the model parameters for probabilistic sensitivity analysis (Table 1). The Monte Carlo simulation used 1 000 iterations to examine the influence of parameter uncertainty on the results.

## Results

### Basic results

For the UK third-party payer, the total costs of anastrozole prevention *vs.* no prevention were £11,470.08 and £7,364.87 with gained 4.594 QALYs and 4.562 QALYs, respectively, and the incremental cost-effectiveness ratio (ICER) was £125,705.38/QALY in the 5-year time horizon model, crosses the threshold of the WTP(£3,000/QALY). In the 12-year time horizon model, the two groups’ costs were £19,154.45 and £15,680.53 and gained 9.909 QALYs and 9.491 QALYs, and the ICER was £8,313.45/QALY (Table 2), less than the WTP.

**Table 2:** The results of cost-effectiveness analysis

Country(time horizon)	Cost	Incremental cost	Utility value(QALY)	Incremental utility value(QALY)	ICER/QALY	WTP threshold	Cost-effective?
***United Kingdom(5 Years)***							
Placebo	£7,364.87	-	4.562	-	-		
Anastrozole	£11,470.08	£4105.20	4.594	0.032	£125,705.38	£ 30,000	No
***United Kingdom(12 Years)***							
Placebo	£15,680.53	-	9.491	-	-		
Anastrozole	£19,154.45	£3473.91	9.909	0.418	£8313.45	£ 30,000	YES
***United States(5 Years)***							
Placebo	$11,671.55	-	4.605	-	-		
Anastrozole	$16,096.51	$4424.96	4.637	0.032	$134,232.13	$ 150,000	YES
***United States(12 Years)***							
Placebo	$23,821.30	-	9.491	-	-		
Anastrozole	$27,516.61	$3695.32	9.909	0.418	$8,843.30	$ 150,000	YES

*ICER* incremental cost-effectiveness ratio, *QALY* quality-adjusted life-year, *WTP* willingness-to-pay

For the third-party payer in the USA, the results showed that in the 5-year time horizon model, the costs of the two groups of anastrozole prevention and no prevention were $16,096.51 and $11,671.55 and gained 4.638 QALYs and 4.605 QALYs, respectively, and the ICER was $134,232.13/QALY. In the 12-year time horizon model, the two groups’ costs were $27,516.61 and $23,821.30 and gained 9.909 QALYs and 9.491 QALYs, and the ICER was $8,843.30/QALY, all less than the WTP threshold ($150,000/QALY) (Table 2). Clearly, anastrozole had advantages in cost-effectiveness for the prevention of breast cancer in high-risk postmenopausal women.

### One-way sensitivity analysis

**Fig. 2:**
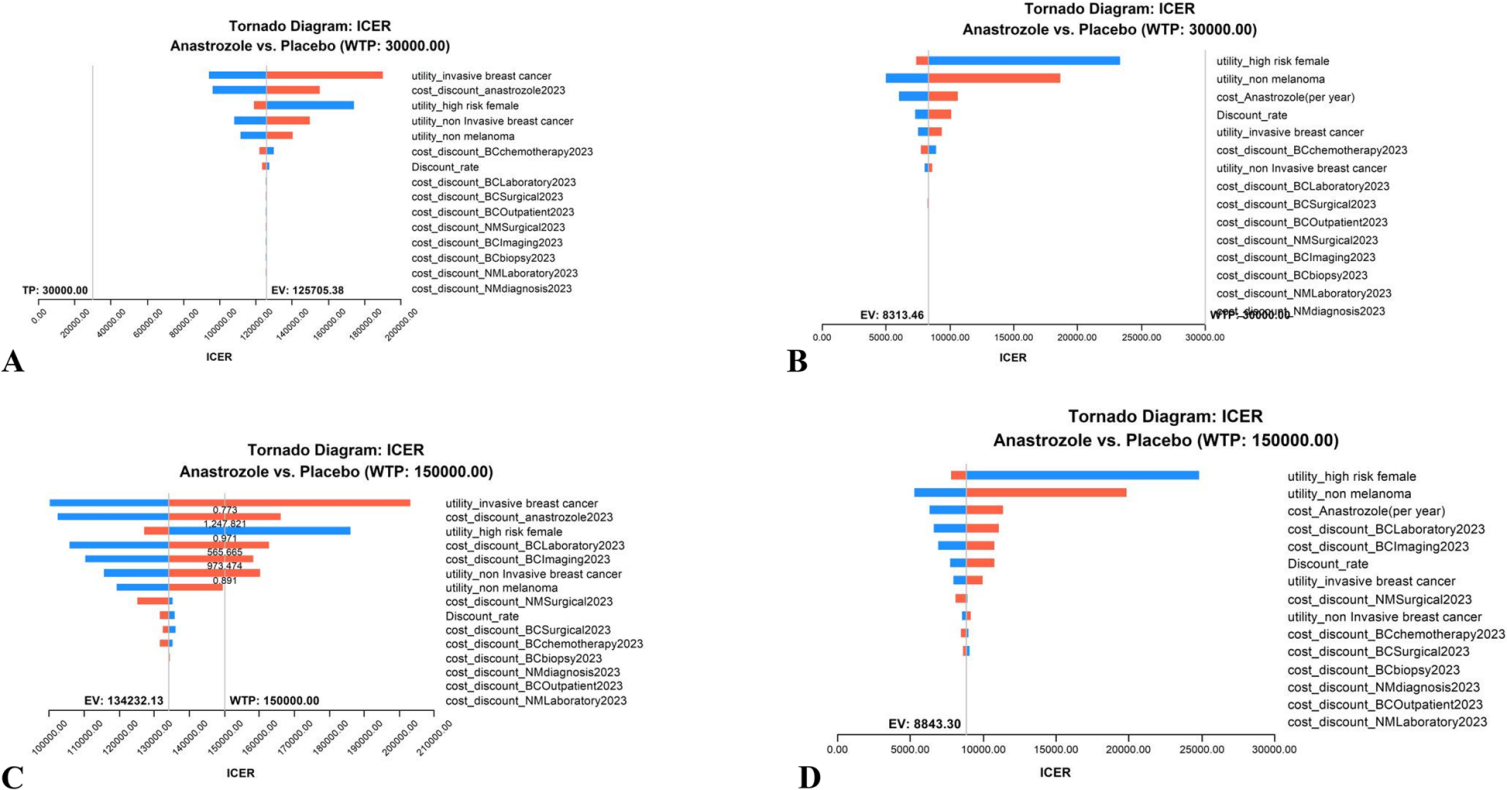
One-way sensitivity analysis for anastrozole prevention versus placebo. A UK of 5-year time horizon model; **B** UK of 12-year time horizon model; **C** USA of 5-year time horizon model; **D** USA of 12-year time horizon model)

The results of the one-way sensitivity analyses are presented in the tornado diagram arranged in order of the degree of influence on the ICER. (Fig. 2). In the first 5 years, the parameters within the range of variation were all above the threshold of the WTP(£3,000/QALY) for the UK. Although the ICER was below the US WTP threshold ($150,000/QALY), the utility of breast cancer, the costs of anastrozole, laboratory assessment and imaging assessment had the greatest impact on the ICER in the US model. However, at the 12-year time horizon, the tornado diagram showed that all variables in the model had no effect on ICER, whether in the UK or US model. The ICERs were also sensitive to the time horizon, particularly in the first 5 years.

### Probabilistic sensitivity analysis

**Fig. 3:**
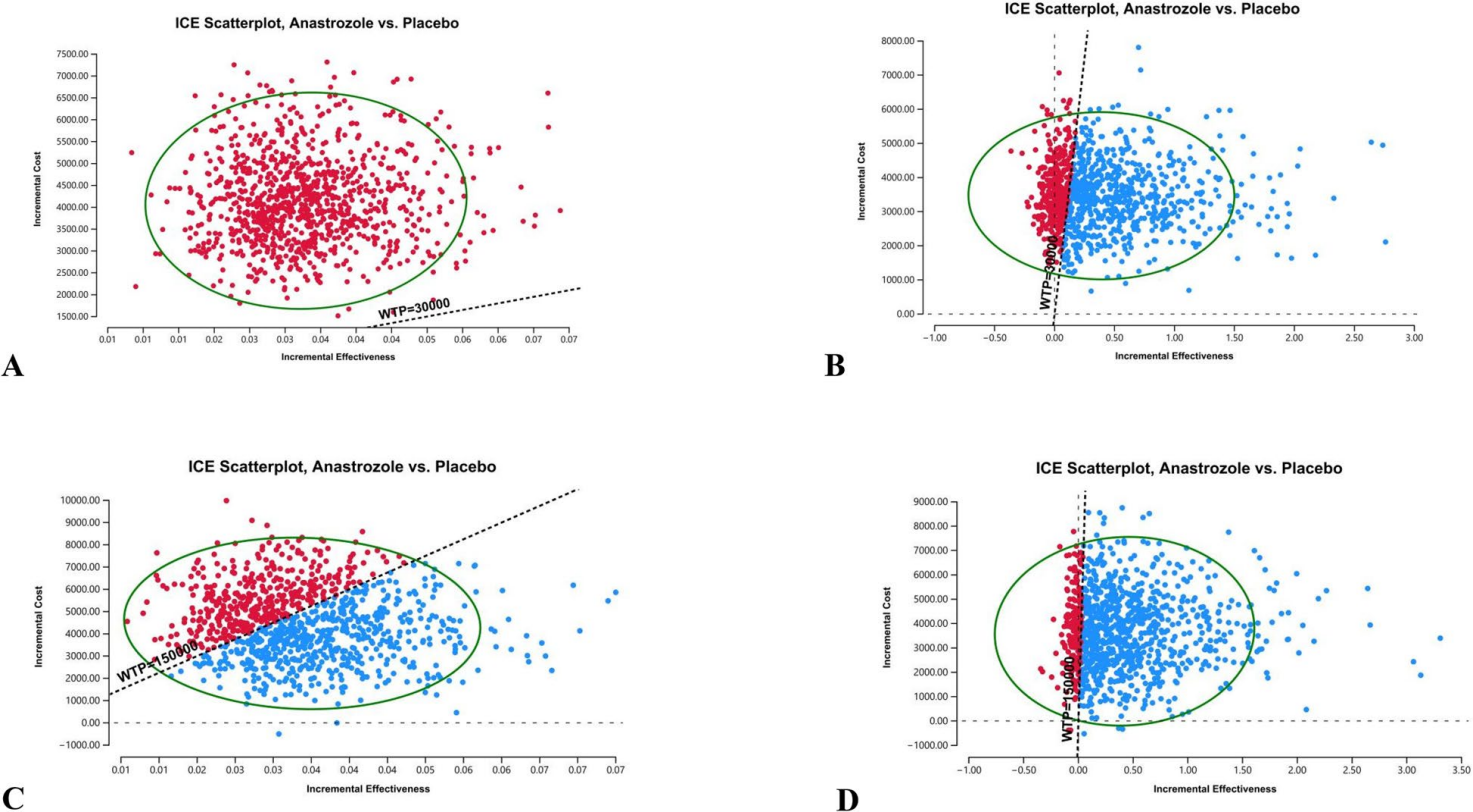
Cost-Effectiveness scattar plot of probabilistic sensitivity analysis for anastrozole prevention versus placebo. **A** UK of 5-year time horizon model; **B** UK of 12-year time horizon model; **C** USA of 5-year time horizon model; **D** USA of 12-year time horizon model)

**Fig. 4:**
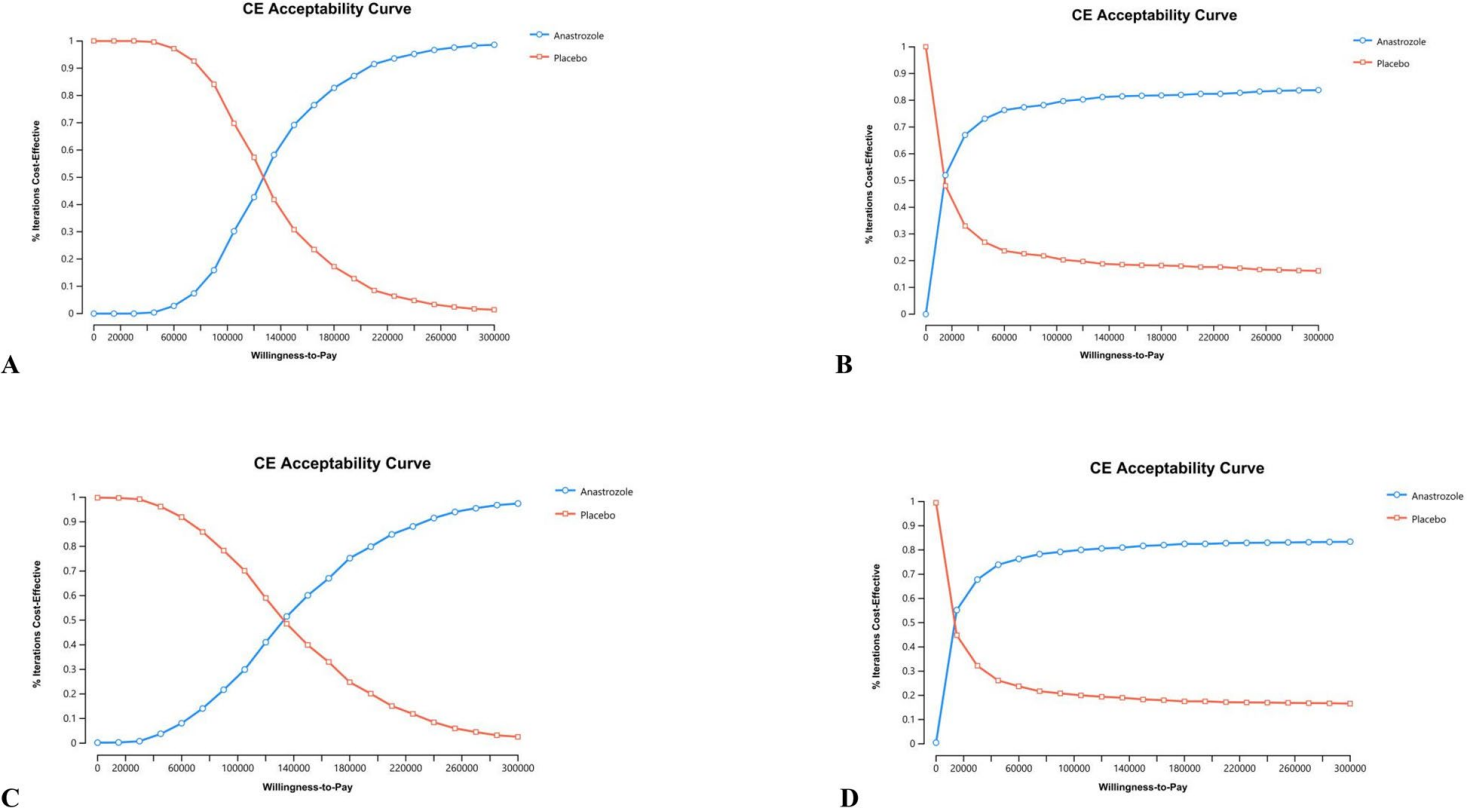
Cost-Effectiveness acceptability curve of probabilistic sensitivity analysis for anastrozole prevention versus placebo. **A** UK of 5-year time horizon model; **B** UK of 12-year time horizon model; **C** USA of 5-year time horizon model; **D** USA of 12-year time horizon model)

The probabilistic sensitivity analysis results are shown in a scatter plot (Fig. 3) and the cost-effectiveness acceptability curve (Fig. 4). The scatter plot indicated that the acceptable proportion of anastrozole prevention for UK was 0% at the £30,000/QALY WTP threshold in the 5-year horizon and approximately 67% in the 12-year horizon. The probability for the USA was approximately 59.9% at the $150,000/QALY WTP threshold in the 5-year horizon and approximately 81.4% in the 12-year horizon. Furthermore, models for 5 and 12 years show that the probability that anastrozole prevention is economical increases with the value of WTP, and the longer the cycle of the model is, the higher the acceptability.

## Discussion

Breast cancer remains the leading cancer-related cause of disease burden for women and is a serious public health concern in high-income countries. Although a reduction in breast cancer risk is important for clinical and treatment outcomes, it is essential to evaluate additional costs to the health care system [36]. Therefore, we created a cost-effectiveness analysis of anastrozole for the prevention of breast cancer in high-risk postmenopausal women based on the IBIS II findings. From the perspective of third-party payers in the UK and the USA, the results of the model suggested that anastrozole increases health care costs but reduces the risk of breast cancer and improves QALYs compared with no prevention. It was a cost-effective advantage of prophylaxis with anastrozole for 12 years. With the extension of the follow-up period, the anastrozole intervention program has a higher economic value. At the same time, in the 5-year-horizon model of the United Stated, we found that the cost of anastrozole is the most important factor affecting the stability of the model, and a higher price of anastrozole may affect the results of ICER, even greater than the WTP threshold, making the whole result uneconomical. In contrast, if its price is lowered, more women at high-risk will benefit. However, in UK economic models, we found a lower acceptance of anastrozole for prevention in high-risk women,which may be related to the lower WTP threshold in the UK. We believe that our findings can provide some reference information for the UK and US healthcare sector.

Currently, the only oestrogen receptor modulators tamoxifen and raloxifene are FDA-approved drugs for breast prevention in postmenopausal women with high risk of breast cancer. After the FDA approved indications for the prevention of these two drugs, there have also been cost-effective studies, especially tamoxifen. Most of the results show that tamoxifen is a cost-effective strategy for preventing breast cancer in high-risk, but most of these trials were done in the 2000s [37], before long-term outcome data on endocrine prophylaxis were available, and the costs of drugs and breast cancer treatment and care have changed over time. Recent results on aromatase inhibitors for breast cancer prevention have shown that AIs have fewer serious adverse events (i.e., endometrial cancer and venous thromboembolism) than tamoxifen or raloxifene, which may offset their higher upfront drug costs. Although none of the AIs are currently FDA approved for breast cancer prevention, many authoritative prevention guidelines, such as NCCN, ASCO, USPSTF, and NICE, recommend them as viable options for the endocrine prevention of breast cancer in postmenopausal women [6]. We also hope that more clinical and economic evidence will help endocrine therapy for the prevention of breast cancer to find a place in routine clinical practice, so that more patients can benefit and the incidence of breast cancer can be reduced.

As we know, this is the first study that focus on the cost-effectiveness of anastrozole for the prevention of breast cancer in high-risk postmenopausal women. However, our present study has several limitations. First, due to the limited follow-up time of the IBS-II study and the lack of PFS and OS survival curves for breast cancer and melanoma, this study used a decision-analytic model to simulate the early progression of the disease and did not consider risk factors for breast cancer or ethnicity of patients. Stratified analysis by ethnicity, age at menopause, body mass index, etc. However, we have carried out sensitivity analysis, and the results show that the model we constructed is relatively robust. Second, our model does not take into account the impact of adverse reactions. Although the study shows that there is no significant difference in adverse reactions between the two groups, adverse reactions will inevitably occur in related treatment and surgery. The progression of diseases is complex and may have an impact on the final outcome. Third, due to differences in economic development, local per capita income and local development GDP are different, and the value of WTP will also be different, so the final results of this study may also be different. Fourth, in low- and middle-income countries(LMICs), due to weak health infrastructure and subsequently poor survival outcomes, prevention for breast cancer remains a challenge [2]. Our study only analysed high-income countries, the UK and the US, and the results cannot be generalised to other LMICs. The cost-effectiveness of anastrozole for the prevention of breast cancer in postmenopausal women at high risk of breast cancer in other countries needs to be further explored.

## Conclusions

In conclusion, this study demonstrates a cost-effective advantage of anastrozole for breast cancer prevention in postmenopausal high-risk women from the perspective of third-party payers in the UK and the USA. The results of this study may provide a reference for the rationality of clinical medication for clinicians and patients and the scientific decision-making of national government departments for medical and health care.

## Data Availability

The datasets supporting the conclusions of this article are included within the article. The link of IBIS-II trial is https://pubmed.ncbi.nlm.nih.gov/31839281/.
